# Hypertrophy of the right ventricle by pulmonary artery banding in rats: a study of structural, functional, and transcriptomics alterations in the right and left ventricles

**DOI:** 10.3389/fphys.2023.1129333

**Published:** 2023-07-27

**Authors:** Jairo Montemor Augusto Silva, Ednei Luiz Antonio, Luis Felipe Neves Dos Santos, Andrey Jorge Serra, Regiane Santos Feliciano, Jose Antonio Silva Junior, Silvia Saiuli Miki Ihara, Paulo Jose Ferreira Tucci, Valdir Ambrosio Moises

**Affiliations:** ^1^ Paulista School of Medicine, Federal University of São Paulo, São Paulo, Brazil; ^2^ Federal University of São Paulo, São Paulo, Brazil; ^3^ Universidade Nove de Julho, São Paulo, Brazil

**Keywords:** pulmonary artery banding, remodeling, hypertrophy, right ventricle, rats

## Abstract

**Introduction:** Right ventricular remodeling with subsequent functional impairment can occur in some clinical conditions in adults and children. The triggering factors, molecular mechanisms, and, especially, the evolution over time are still not well known. Left ventricular (LV) changes associated with right ventricular (RV) remodeling are also poorly understood.

**Objectives:** The study aimed to evaluate RV morphological, functional, and gene expression parameters in rats submitted to pulmonary artery banding compared to control rats, with the temporal evolution of these parameters, and to analyze the influence of RV remodeling by pulmonary artery banding in rats and their controls over time on LV geometry, histology, gene expression, and functional performance.

**Methods:** Healthy 6-week-old male Wistar-EPM rats weighing 170–200 g were included. One day after the echocardiogram, depending on the animals undergoing the pulmonary artery banding **(**PAB) procedure or not (control group), they were then randomly divided into subgroups according to the follow-up time: 72 h, or 2, 4, 6, or 8 weeks. In each subgroup, the following were conducted: a new echocardiogram, a hemodynamic study, the collection of material for morphological analysis (hypertrophy and fibrosis), and molecular biology (gene expression). The results were presented as the mean ± standard deviation of the mean. A two-way ANOVA and Tukey post-test compared the variables of the subgroups and evolution follow-up times. The adopted significance level was 5%.

**Results:** There was no significant difference among the subgroups in the percentage of water in both the lungs and the liver (the percentage of water in the lungs ranged from 76% to 78% and that of the liver ranged from 67% to 71%). The weight of the right chambers was significantly higher in PAB animals in all subgroups (RV PAB weighed from 0.34 to 0.48 g, and control subjects, from 0.17 to 0.20 g; right atrium (RA) with PAB from 0.09 to 0.14 g; and control subjects from 0.02 to 0.03 g). In the RV of PAB animals, there was a significant increase in myocyte nuclear volume (97 μm^3^–183.6 μm^3^) compared to control subjects (34.2 μm^3^–57.2 μm^3^), which was more intense in subgroups with shorter PAB follow-up time, and the fibrosis percentage (5.9%–10.4% vs. 0.96%–1.18%) was higher as the PAB follow-up time was longer. In the echocardiography result, there was a significant increase in myocardial thickness in all PAB groups (0.09–0.11 cm compared to control subjects–0.04–0.05 cm), but there was no variation in RV diastolic diameter. From 2 to 8 weeks of PAB, the S-wave (S’) (0.031 cm/s and 0.040 cm/s), and fractional area change (FAC) (51%–56%), RV systolic function parameters were significantly lower than those of the respective control subjects (0.040 cm/s to 0.050 cm/s and 61%–67%). Furthermore, higher expression of genes related to hypertrophy and extracellular matrix in the initial subgroups and apoptosis genes in the longer follow-up PAB subgroups were observed in RV. On the other hand, LV weight was not different between animals with and without PAB. The nuclear volume of the PAB animals was greater than that of the control subjects (74 μm^3^–136 μm^3^; 40.8 μm^3^–46.9 μm^3^), and the percentage of fibrosis was significantly higher in the 4- and 8-week PAB groups (1.2% and 2.2%) compared to the control subjects (0.4% and 0.7%). Echocardiography showed that the diastolic diameter and LV myocardial thickness were not different between PAB animals and control subjects. Measurements of isovolumetric relaxation time and E-wave deceleration time at the echocardiography were different between PAB animals and control subjects in all subgroups, but there were no changes in diastolic function in the hemodynamic study. There was also increased expression of genes related to various functions, particularly hypertrophy.

**Conclusion:** 1) Rats submitted to pulmonary artery banding presented RV remodeling compatible with hypertrophy. Such alterations were mediated by increased gene expression and functional alterations, which coincide with the onset of fibrosis. 2) Structural changes of the RV, such as weight, myocardial thickness, myocyte nuclear volume, and degree of fibrosis, were modified according to the time of exposure to pulmonary artery banding and related to variations in gene expression, highlighting the change from an alpha to a beta pattern from early to late follow-up times. 3) The study suggests that the left ventricle developed histological alterations accompanied by gene expression modifications simultaneously with the alterations found in the right ventricle.

## 1 Introduction

Pressure overload on the right ventricle (RV) occurs in congenital heart diseases, such as those associated with obstruction of the infundibulum, pulmonary valve, trunk, and pulmonary branches (Baumgartner et al., 2010), and in the increase of pulmonary pressure after left ventricular dysfunction, mitral valve disease, chronic obstructive pulmonary disease (COPD), and pulmonary arterial hypertension (Faber et al., 2006; Koletyis et al., 2007). In situations with a mild or moderate increase in the systolic pressure of the RV, the repercussions are usually not significant in the short term; however, in the long term, expressive alterations can occur. In situations with an expressive increase in the systolic pressure of the RV, the remodeling process can be faster and reach the late stages of difficult functional recovery (Leeuwenburgh et al., 2003; Faber et al., 2006; Peter and Glenn MarsboomJanssens, 2007; Bogaard HarmJ. et al., 2009).

RV remodeling occurs due to the necessity of generating greater strength to overcome the outflow tract obstruction or increased pulmonary pressure. In parallel, a replication of sarcomeres determines the increase in the ventricular mass and decreases the parietal stress (Laplace’s law), compensating for the increased blood pressure demand and allowing the initial stabilization of the cardiac function. Such changes are progressive and deleterious, determining the unfavorable evolution of the RV, which becomes unable to generate a higher pressure and keep the debit, with the subsequent development of heart failure (HF) (Peter and Glenn MarsboomJanssens, 2007; Kittipovanonth et al., 2008). The RV remodeling and left ventricle (LV) result in intracellular gene activation and repression mechanisms that encode certain proteins (Rockman et al., 1994; Faber et al., 2005; Qipshidze et al., 2012). Remodeling includes myocyte hypertrophy, necrosis, and apoptosis, along with changes in collagen and interstitial fibrosis, and it has been studied in animal models (Binkhorst et al., 1989; Binkhorst et al., 1989; Rockman et al., 1994; Faber et al., 2005; Urashima et al., 2008). Several genes and proteins involved were identified, and such information can be related to the anatomic and functional parameters measured by the echocardiogram and hemodynamic study at different stages of remodeling (Binkhorst et al., 1989; Rockman et al., 1994; Urashima et al., 2008; Witt et al., 2008; Qipshidze et al., 2012; Drake et al., 2023).

LV remodeling has more widely been studied than RV remodeling. The majority of animal models used are rats and mice.

Animal models to study the remodeling of RV aim to increase the post-load using a partial obstruction (banding) of the pulmonary artery (PAB) or the increase of pulmonary pressure caused by monocrataline, hypoxia, or COPD via cigarette smoke intake with repeated infections (Faber et al., 2006; Urashima et al., 2008; Baandrup et al., 2011; Li et al., 2013; Li et al., 2013).

The cerclage model, or partial pulmonary artery constriction, is more appropriate. The hypertrophy degree appears to relate to the severity of the obstruction, and the more prominent histological changes appear to occur in the more prolonged obstructions (Schou et al., 2007; Qipshidze et al., 2012).

Experimental studies on overload on the RV denote that the increase in the production of collagen and fibrosis determines an equivalent increase in the reactive oxygen species (ROS), causing oxidative damage to mitochondria (Baandrup et al., 2011). At the same time, there is an increase in gene expression involved in oxidative stress, mitochondrial metabolism, myocardial hypertrophy, and calcium and phosphorus kinetics (Baandrup et al., 2011). Furthermore, apoptotic pathways related to the mitochondria are activated, which favors ventricular remodeling (Urashima et al., 2008; Qipshidze et al., 2012). Another source of data that suggests adverse ventricular adaptation is the increase in the expression of fetal genes due to the activation of transcription factors in the nucleus, such as Nfat, calcitonin, N-Meft, or calmodulin, which depend on the protein kinase CaMK or mitogen kinase (Frey and Olson, 2003; Morisco et al., 2003).

Gene expression analysis on RV overload can be conducted using quantitative RT-PCR assays in order to identify multiple targeted genes (Faber et al., 2006; Kitahori et al., 2009). Although this technique does not allow for a comprehensive analysis of gene expression, as do microarrays, its limitations can be overcome by properly choosing the targeted genes. The data available in the literature allow, in part, to determine the evolutionary relation between the gene expression and the morphometric, histological, or functional aspects of RV, with simultaneous studies of RV and LV alterations, as proposed in this model. Deeper knowledge of this relationship may improve the understanding of how gene expression changes induce the different types of RV remodeling under pure pressure overload and the consequences on the LV.

A Doppler echocardiogram has been performed in animal models of RV hypertrophy (Lang et al., 2005; Faber et al., 2006; Hardziyenka et al., 2006; Horton et al., 2009; Rudski LG. et al., 2010; Rudski et al., 2010b). The more widely used variables are the systolic and diastolic areas of the cavity as indicators of dilatation and systolic function (Schou et al., 2007; Rudski LG. et al., 2010). Studies have also noted that the pressure gradient measured by Doppler echocardiography through the PAB relates to RV pressure (Faber et al., 2006). Other possible variables to be assessed through an echocardiogram, such as RV wall thickness and other indexes of systolic wave (S’) and diastolic function, have not been fully analyzed and compared to the standards of histology, morphometry, and catheterization by published studies (Lang et al., 2005; Hardziyenka et al., 2006; Rudski LawrenceG. et al., 2010).

It is understood that both ventricles have common characteristics and depend on electric, hemodynamic, and humoral activation (Leeuwenburgh et al., 2003; Faber et al., 2006; Peter and Glenn MarsboomJanssens, 2007; Bogaard HarmJ. et al., 2009). Although the repercussions of LV dysfunctions in RV remodeling are relatively known, there is scarce information on the effects of RV changes on the LF structure and function. Findings in COPD and pulmonary emphysema patients illustrate a reduction of the ejection fraction of the LV (Gottdiener et al., 1985; Noordegraaf AV. et al., 1997). Systolic changes in LV in dogs bearing pulmonary emphysema were also observed (Laks et al., 1972).

This study was conducted to verify the effects of pulmonary artery banding on the structure, function, and gene expression of the right and left ventricles of the rat heart. The study took measurements at various time points over an 8-week period to understand the evolution of ventricular remodeling and identify transitions from a physiological adaptive process to a state of maladjustment. In doing so, we intend to identify the appropriate moment to implement therapy and prevent maladaptive remodeling.

## 2 Materials and methods

### 2.1 Animals

A total of 151 male Wistar-EPM rats of 6 weeks of age (170–200 g) were included. The study sample size was defined similarly as in previous studies using variance analysis (ANOVA) with a test power of 80% and an alpha level of 0.05 (Rockman et al., 1994; Faber et al., 2005).

### 2.2 Experimental design

**FIGURE 1:**
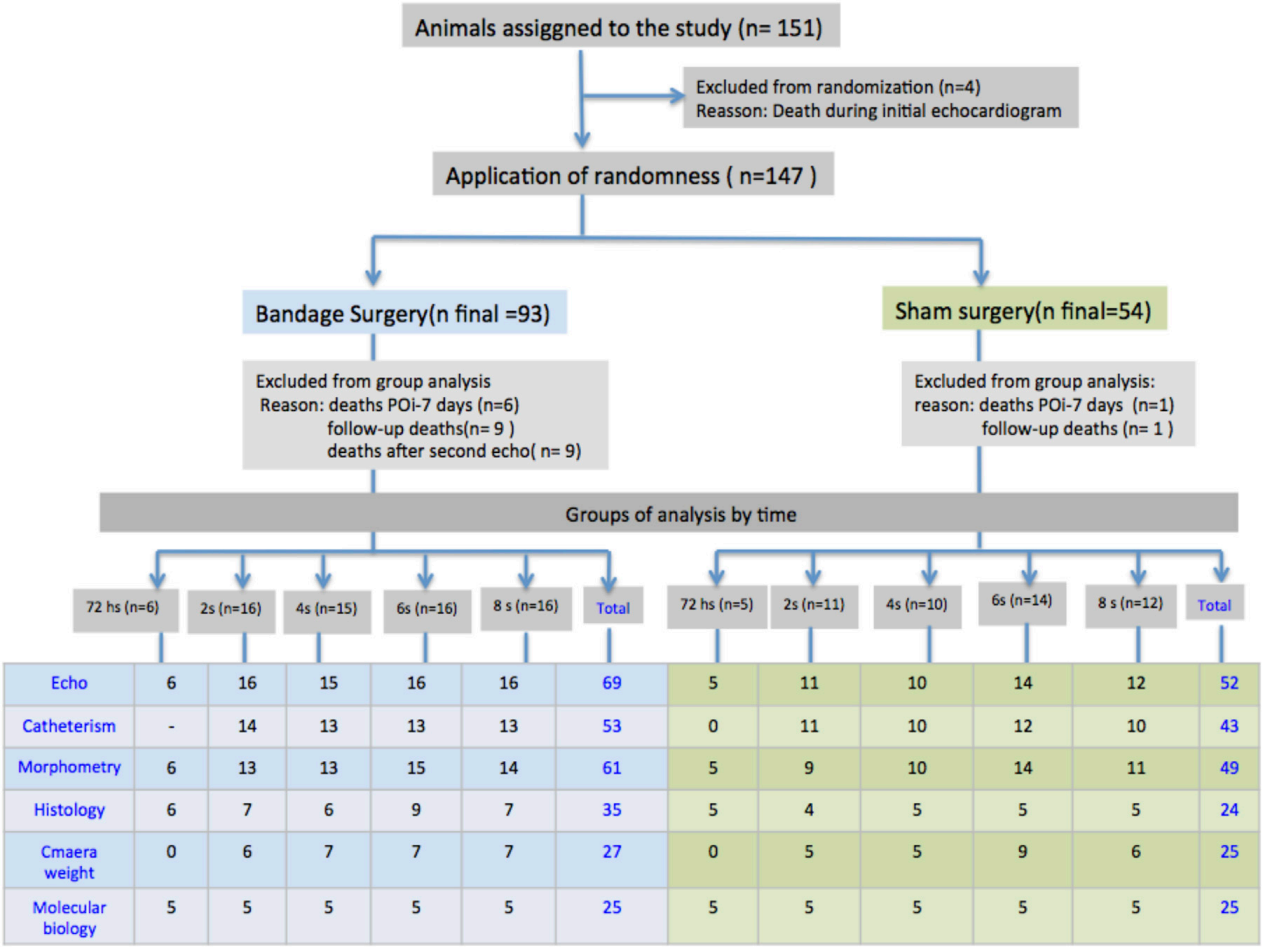
Schematic layout of the procedures, occurrences, and distribution of the animals of the study in the groups with PAB and control subjects, and the respective evolution subgroups; N = number; POi, immediate postoperative period; S, weeks; Sham, control.

Initially, the animals were submitted to the Doppler echocardiogram after performing PAB or simulated surgery (control group). On the third day following the surgical procedure, the animals were distributed randomly into five subgroups according to the follow-up time of 72 h, or 2, 4, 6, and 8 weeks in such a way that each subgroup included both control animals and animals with bandages (Figure 1). The analysis periods were collected by representing the infancy and adolescence of these animals; 8-week-old animals are young adults. On the determined date for each subgroup closure, another echocardiogram was performed, followed by a hemodynamic study and material collection for histological and molecular biology analyses (Figure 1).

### 2.3 Pulmonary artery banding (PAB)

The PAB method in the animals followed that used by Rockman et al. (1994), de Melo et al. (2016). The animals were anesthetized with halothane, placed in dorsal decubitus, intubated, and maintained on mechanical ventilation with positive pressure in a ventilator for rodents (model Harvard 683, Holliston, MA, United States). After trichotomy of the left hemithorax, an incision was made in the skin, using tweezers and scissors, in the mid-axillary line, a finger below the armpit. With the aid of curved scissors, the pectoral muscles were pulled apart, and with hemostatic forceps, the intercostal muscles were divulsed; two “Stevenson” type surgical retractors were placed in the intercostal space to spread the ribs and enlarge the thoracotomy. The animal’s left front paw was released from the original position of dorsal decubitus and fixed near the right front paw to position the thorax in the right lateral decubitus (30°), favoring the exposure of the heart base. After separating the aorta, the pulmonary artery trunk was tied with a cotton thread along with a 1.3-mm catheter positioned in parallel. This catheter was removed after fixing the loop. After that, with the heart in its original position, lung hyperinflation was performed with concomitant closure of thoracotomy by suturing in a bag previously prepared around the incision. After this procedure, the animal was kept on mechanical ventilation enriched with oxygen until spontaneous ventilation occurred. After respiratory stabilization, the ventilatory support was removed and the animal was placed in a heated plastic box during the postoperative period until complete post-anesthetic recovery under supervision. The control animals (sham surgery) were submitted to the same procedures except for the fixation of the loop in the pulmonary artery trunk.

### 2.4 Echocardiogram

A complete Doppler echocardiogram was performed on all study animals before randomization. A second echocardiogram, also complete, was performed on all animals at the end of each observation period, according to randomization at 72 h or 2, 4, 6, and 8 weeks. The animals were anesthetized for echocardiography with a mixture of ketamine hydrochloride (50 mg/kg) and xylazine hydrochloride (10 mg/kg), followed by trichotomy of the anterior and lateral left thorax. The animals were positioned in left lateral decubitus, and three electrodes were placed in the paws to obtain the electrocardiographic stroke simultaneously with the echocardiographic image. The examination was performed according to a technique already established in our laboratory with a Philips SONOS 5500^®^ HP device (Philips Medical System, Andover, Mass., United States) with a 5–12-MHz transducer that allows real-time cardiac imaging in two-dimensional and one-dimensional (M-mode) modes (Moisés et al., 2000; Nozawa et al., 2006) and analysis with the different Doppler techniques. The images were obtained through the parasternal windows with the cuts (transverse and longitudinal), and apical four and five chambers. The examination was completed with flow velocity analysis by color flow mapping, spectral and continuous Doppler, and tissue Doppler imaging. The images were recorded on videotapes for later measurement and analysis, of the anatomical and functional parameters. For morphological and functional analysis of the heart, several measurements were performed, and various parameters were calculated according to previous experience of our laboratory and international guidelines with adaptations for the animals of the present study (Moisés et al., 2000; Cury et al., 2005; Lang et al., 2005; Hardziyenka et al., 2006; Nozawa et al., 2006; Urashima et al., 2008; Nagueh et al., 2009; Rudski LawrenceG. et al., 2010). Descriptions of all measurements can be found in Supplementary Annex S1 at the end of this text.

### 2.5 Hemodynamic study

The hemodynamic study was conducted with the animals under anesthesia adjusted with urethane (1.2 g/kg, i.v.) and one micromanometer Millar (Mikro-Tip^®^ 2F, Millar Instruments Inc., Houston, TX, United States), which had its distal end positioned inside the left ventricular cavity from the catheterization of the right carotid artery to measure intraventricular pressure. The right chambers were reached through a venous catheter with a pressure transducer. The data obtained were analyzed using AcqKnowledge^®^ 3.7.5 (BIOPAC Systems Inc., CA, United States), which measures instantaneous values of systolic ventricular pressures (PVS) and final diastolic pressure (RVEDP), both expressed in mmHg heart rate in beats per minute (bpm) and the first-time derivative of positive (dP/dT, in mmHg/s) and negative pressure (-dP/dT, in mmHg/s).

### 2.6 Macroscopic and histological analyses

The material for this analysis was randomly obtained from the hearts of the animal sample at the end of each observation period according to the randomization of 72 h or 2, 4, 6, and 8 weeks.

Subsequent to the hemodynamic study, the supplementation of anesthesia with intraperitoneal 4.8 g/kg. Then, animals were subjected to total thoracotomy and a ventricular retrograde perfusion was performed via the aorta. Afterward, the superior and inferior vena cava were clamped, and a small incision drained the atria to prevent the blood from these cavities from reaching the ventricles; a phosphate buffer solution was infused (for 1 min) in order to remove the blood residue. Subsequently, a 10% buffered formalin solution was infused for 10 min until it reached the coronary circulation and promoted a greater tissue fixation. After this preparation, the hearts were removed and cut transversally in the middle region of the ventricles, photographed, and stored in previously identified individual flasks containing 10% formaldehyde for 72 h. These flasks were sent to the pathological anatomy laboratory, where they were dehydrated in increasing concentrations of ethyl alcohol, diaphonized in xylene, and embedded in paraffin. After that, 4-μm tissue sections were made in the paraffin blocks using a Minot microtome (Leica RM 2035), collected on glass slides (two of both techniques for each animal), and kept at 37°C for drying, bonding, and subsequent staining by the hematoxylin–eosin (HE) method and the picrosirius method. For the quantitative analysis through histology, the slides were prepared by hematoxylin–eosin staining (HE), and picrosirius included measures of the nuclear volume of the myocytes and the myocardial collagen content (Serra et al., 2010).

### 2.7 Determination of heart weight and heart chamber weight

After anesthesia with a high dose of urethane, the animals’ hearts assigned for molecular biology studies were removed without the base vessels. Initially, the total weight of the hearts was obtained, and after the separation, the four heart chambers were weighed in isolation; the ventricular septum was included with the LV.

### 2.8 Lung and liver water contents

Pulmonary and systemic tissue congestions were measured, respectively (Nozawa et al., 2006; Veiga et al., 2019). After euthanizing the animals, the right lung was isolated by stitching cotton thread around the pulmonary hilum. A similar procedure was performed with part of the central lobe of the liver. Both were removed from the animals and immediately weighed to obtain the wet weight (PU). Subsequently, these tissue samples were kept in a drying and sterilizing oven for 12 h, followed by another weighing to obtain the dry weight (PS) (Garry and Olson, 2006; Nozawa et al., 2006). Lung and liver water contents (%H_2_O) were calculated according to the formula %H_2_O = [(PU–PS)/PU] × 100; %H_2_O is the water content.

### 2.9 Targeted gene expression

Gene expression was analyzed through RT-PCR and targeted at the targets involved in RV remodeling, resulting from pressure overload. As illustrated in Table 1, (Gottdiener et al., 1985) genes were selected (Hosenpud et al., 1989; Amanuma et al., 1994; Thaik et al., 1995; Thaik et al., 1995; Olivetti et al., 1997; Kang and Izumo, 2000; Hein et al., 2003; Kögler et al., 2003; Morisco et al., 2003; Giordano, 2005; Roncon-Albuquerque et al., 2006; Urashima et al., 2008; Correia-Pinto et al., 2009; Saini-Chohan et al., 2011; Fang et al., 2012; Egemnazarov et al., 2015; Zile and Gregg, 2016; Drake et al., 2023). In the animals of the 72-h subgroup, only the RV was analyzed, and in the other subgroups, the tissues of both ventricles were analyzed.

**TABLE 1:** Genes analyzed according to the associated function.

Function	Genes		
Control subjects	*GAPDH*	18S	
Extracellular matrix	*Col3a1*	*Mnp9*	*Tgf*
	*TNC*	*Col1a1*	*Map3K2*
Hypertrophy	*IGF1*	*Cabin1*	*ACE Edn1*
	*Chp2*	*Ace2*	*Nppa*
	*PrKcb*	*Nppb*	*Nfatc3*
	*Agr1a*	*PrKcg*	*PrKca*
Angiogenesis	*Vegfa*		
Oxidative stress	*GPX4*	*Hspa1a*	*CAT*
	*Sod1*		
Apoptosis	*Bax*	*MapK14*	*Mapk1*
	*Faz*	*Tp53*	*AKt1*
Calcium kinetics	*Pin*	*Slac8a1*	*Atp2a2*
	*Casq2*	*Ryr2*	
Cellular metabolism	*Hk1*	*Ndufa3*	*Ucp2*
	*Taz*	*Pfkm*	*Slc2a1*
Myofibrillar protein (fetal)	*Myh6*	*Myh7*	
Inflammation	*Tnf*	*Tnfrsfla*	*IL6*

References: Hosenpud et al. (1989); Amanuma et al. (1994); Thaik et al. (1995); Olivetti et al. (1997); Kang and Izumo (2000); Hein et al. (2003); Kögler et al. (2003); Morisco et al. (2003); Giordano (2005); Roncon-Albuquerque et al. (2006); Urashima et al. (2008); Correia-Pinto et al. (2009); Saini-Chohan et al. (2011); Fang et al. (2012); Zile and Gregg (2016); Drake et al. (2023)]; Egemnazarov et al. (2015).

### 2.10 RNA extraction

The heart samples from both ventricles, weighing 0.2 and 0.5 g, were homogenized in the TRIzol^®^ reagent for total RNA extraction according to the manufacturer’s guidelines. The total RNA extracted was treated with 10 U of desoxyribonuclease (RNase)-free for 1 h at 37°C. Afterward, the extraction was performed with an equal volume mixture containing phenol–chloroform–isoamyl alcohol in the proportion 25:24:1, followed by precipitation with 0.2 M sodium acetate and two volumes of absolute ethanol. The precipitated RNA was washed with 70% ethanol to eliminate phenol and salt residue, and solubilized in water. Sample integrity was verified by agarose gel electrophoresis at 1%, containing 0.5 μg/mL of ethidium bromide.

### 2.11 Reverse transcription (RT)

For synthesizing cDNA (complementary DNA), 5 μg of RNA total was used. The samples were incubated with 0.5 μg/mL of oligo dT at 65°C for 5 min to obtain the first tape of cDNA 12–18. RT was conducted with a total volume of 20 μL containing 10 mM of dNTPs, 0.1 M of DTT, 1X enzyme buffer, 3 U of RNA, and 2.5 U of reverse transcriptase (AMV-RT). After incubation for 1 h at 37°C, the temperature was increased to 95°C for 5 min, and the samples were rapidly placed on ice for denaturation of the RNA–cDNA hybrids formed and inactivation of the enzyme used in the reaction. In some tubes, reverse transcriptase was not added to control contamination or amplification of genomic DNA. The obtained cDNA was stored at −20°C for future conduction of the PCR reaction.

#### 2.11.1 Polymerase chain reaction

The cDNA in the stock obtained at the previous stage was used as a mold in the PCR reactions to avoid contamination by genomic DNA. Primes that detect specific molecules of non-transcribed genomic DNA were used, following the manufacturer’s guidelines. After PCR, 10 µL of each sample was submitted to agarose gel electrophoresis at 1% to detect contamination of genomic DNA. The amplification products were identified according to their molecular weight.

### 2.12 Quantitative PCR array

An “RT2 Profiler™ PCR Array–Signal Transduction Pathway Finder” set (SuperArray Bioscience Corporation^®^, United States) was used for this reaction. Amplification, data acquisition, and melting were performed through one iCycler iQ™ thermocycler (Bio-Rad Laboratories^®^). The amplification reactions for targeted genes were performed using an SYBR Green probe in PE Applied Biosystems equipment (ABI Prism 7000). The software iCycler iQ™ version 3 (Bio-Rad Laboratories^®^) was used for data processing. The expressed genes were normalized by the level of expression of housekeeping genes *GAPDH* or 18Sr RNA in the data analyzed by ABI PRISM 7000 Sequence Detection System version 1.6 software.

### 2.13 Statistical analysis

The data were analyzed using SPSS (version 13.0) software, and the statistical significance level adopted was 5%. The quantitative variables were presented as mean ± standard deviation. The Kolmogorov–Smirnov test was applied to verify the normal distribution of data.

The comparison of variables between the PAB and control groups in every subgroup was performed by the *t*-test when the distribution was normal or by the Wilcoxon test when it was not normal. The comparison between subgroups (different PAB follow-up times) of the morphological, echocardiographic, and molecular variables was performed using the two-way ANOVA when the distribution was normal, complemented by the Tukey post-test or the Kruskal–Wallis test complemented by Dunn’s post-test when the distribution was not normal.

## 3 Results

A total of 147 Wistar rats were subjected to PAB surgery or a simulated procedure (control group) (Figure 1). Of these, 30 animals (19.9%) died. The deaths occurred at the following times: four during the first echocardiogram before randomization, seven between surgery and the 7th postoperative period, of which six had undergone PAB, and one was in the control group, 10 in the follow-up period, of which nine were from the PAB group, and one was from the control group, five related to the second echocardiogram in the group subjected to PAB, and four related to invasive hemodynamic study also in the group subjected to PAB. Therefore, 121 animals were included; due to the study design, the number of animals varied according to the analysis.

Wet and dry weight, and liver and pulmonary water content percentages were not significantly different among the experimental groups (Table 2).

**TABLE 2:** Means and standard deviations of total and dry weight (g), percentages of water in part of the liver and central lobe of the right lung, and comparison between animals with and without BAP and between subgroups of animals with BAP over time.

Subgroup	Liver (g)	Liver dry	% liver water	Lung (g)	Dry lung	% lung water
BAP 72 hs	1.5 (±0.2)	0.41 (±0.1)	71.7 (±0.8)	1.1 (±0.1)	0.24 (±0)	76 (±0.8)
Control 72 hs	1.62 (±0.2)	0.46 (±0.1)	71.2 (±0.4)	1.1 (±0)	0.25 (±0)	76 (±0)
p	ns	ns	ns	ns	ns	ns
BAP 2 w	1.1 (±0.3)	0.3 (±0.1)	68.2 (±10)	1.21 (±0.2)	0.26 (±0)	78 (±0)
Control 2 w	1.1 (±0.2)	0.31 (±0.1)	69.7 (±0.9)	1.4 (±0.3)	0.3 (±0.1)	77 (±0)
p	ns	ns	ns	ns	ns	ns
BAP 4 w	1.2 (±0.5)	0.34 (±0.1)	71 (±2.3)	1.2 (±0.5)	0.28 (±0.1)	7 (±0.2)
Control 4 w	1.1 (±0.1)	0.33 (±0.03)	69.8 (±0.8)	1.5 (±0.2)	0.30 (±0)	78 (±0)
p	ns	ns	ns	ns	ns	ns
BAP 6 w	1.29 (±0.33)	0.38 (±0.1)	70 (±1.5)	1.2 (±0.1)	0.27 (±0)	77 (±0)
Control 6 s	1.31 (±0.1)	0.42 (±0.1)	67.3 (±6.1)	1.4 (±0.2)	0.3 (±0)	77 (±0)
p	ns	ns	ns	ns	ns	ns
BAP 8 w	1.3 (±0.23)	0.38 (±0.1)	70 (±2.9)	1.4 (±0.3)	0.3 (±0.1)	77 (±0)
Control 8 w	1.32 (±0.44)	0.39 (±0.1)	69.9 (±0.8)	1.3 (±0.1)	0.3 (±0)	78 (±0)
p	ns	ns	ns	ns	ns	ns

BAP, pulmonary artery banding; hs, hours; ns, non-significant difference; p, statistical significance by a t-test between animals with pulmonary artery banding and controls in each subgroup; w, weeks.

**FIGURE 2:**
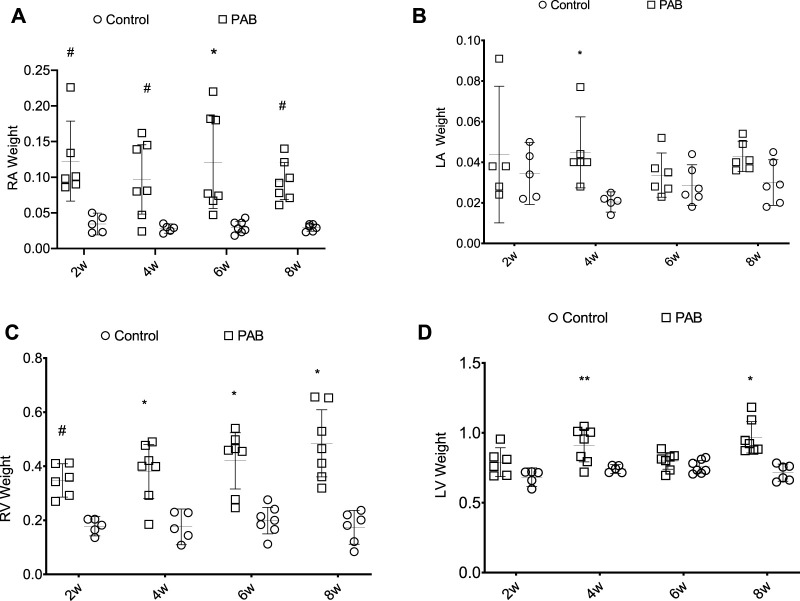
Weight distribution (g) of the cardiac chambers in PAB animals and control subjects in the different follow-up times of analysis; w, weeks. *p < 0.001; **p < 0.01; #*p* < 0.05; RA, right atrium; LA, left atrium; RV, right ventricle; LV, left ventricle.

Regarding the myocardial mass, RA weight was significantly higher in PAB animals than their respective control subjects at all times of the study (Figure 2A). There was no significant difference between the PAB subgroups. RV weight in PAB animals was significantly greater in all subgroups compared to the control subjects (Figure 2C), and a greater RV weight was found in PAB animals at 8 weeks compared to 2 weeks. LA weight of the animals with PAB was significantly higher than that of the control subjects at only 4 weeks (Figure 2B); there was no significant difference among the PAB subgroups. The LV weight of the PAB animals was significantly greater than the control subjects at 4 and 8 weeks. A greater LV weight among PAB animals was identified compared to 4 and 8 weeks, and 2 and 6 weeks (Figure 2D).

**FIGURE 3:**
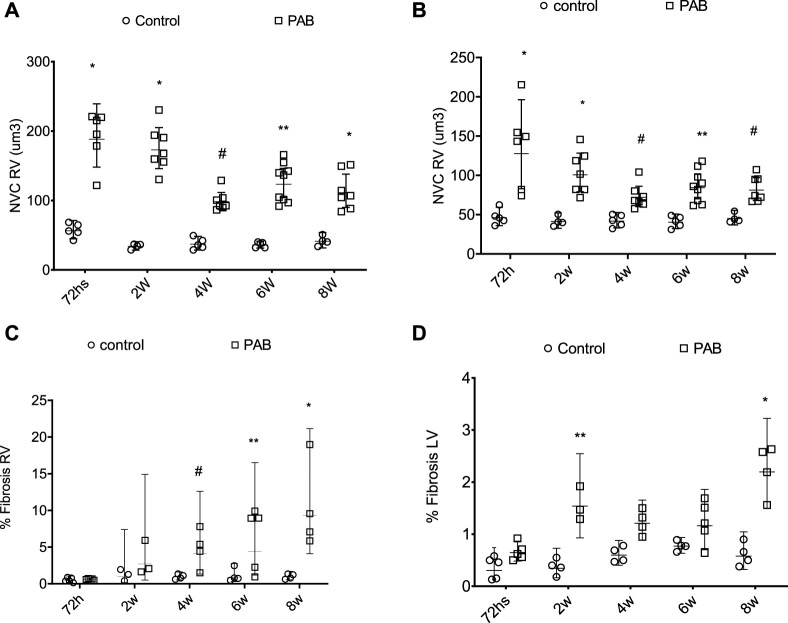
Behavior of the nuclear volume of the cardiomyocytes (NVC) above **(A,B)** and fibrosis percentage (%) below **(C,D)** of the right ventricle (RV) and left ventricle (LV) in PAB animals and control subjects. *p < 0.001; **p < 0.01; #*p* < 0.05.

The volume of the nucleus of the myocytes of both RV and LV was significantly greater in PAB animals compared to the control subjects over all the study follow-ups (Figures 3A, B). For the RV of PAB animals, the nuclear volume was significantly greater at 72 h compared to 4, 6, and 8 weeks and at 2 weeks compared to 4, 6, and 8 weeks. The nuclear volume of the LV in the 72-h PAB group was significantly greater than that in the other groups, and at 2 weeks, it was significantly different from that at 4 weeks.

The fibrosis percentage in the RV was significantly higher in PAB animals than the control subjects at 4, 6, and 8 weeks (Figure 3C). For PAB animals, the fibrosis percentage was significantly higher at 4, 6, and 8 weeks than at 72 h and at 8 weeks than at 2 weeks. The fibrosis percentage in the LF was significantly higher in PAB animals than that of the control subjects at 2 and 8 weeks (Figure 3D). In the PAB subgroups, there was no significant difference in the nuclear volume and the fibrosis percentage of both ventricles among the different subgroups.

**FIGURE 4:**
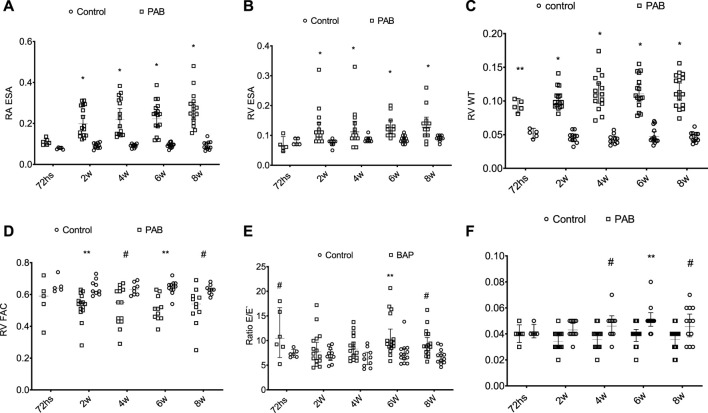
Behavior of the anatomic parameters of the right cavities and systolic function of the right ventricle during the echocardiogram of PAB animals and control subjects. ESA, end systolic area (cm^2^); RA, right atrium; E/E’ ratio; RV WT, diastolic thickness of the free wall of the RV (cm); RV, right ventricle; FAC, fractional area change; S’, systolic displacement speed of the side wall of the RV. W, weeks; *p < 0.001; **p < 0.01; #*p* < 0.05.

During the echocardiogram, the heart rates of PAB subgroup animals were not significantly different from those of the control subjects. In the analysis of the parameters of the right chambers, we found that RA end-systolic area (ESA) and RV ESA of the PAB subgroup were significantly higher than those in the control subjects at 2, 4, 6, and 8 weeks (Figures 4A, B). The RV wall thickness (RV WT) of PAB rats was significantly higher than that of the control subjects in all subgroups (Figure 4C). Among the systolic function parameters of the RV, TAPSE was significantly lower in PAB animals than in the control subjects at 72 h and FAC and S wave were significantly higher than those in the controls in subgroups at 2, 4, 6, and 8 weeks (Figures 4D, F).

In the PAB subgroup animals, RA ESA and RV ESA were significantly higher at weeks 2, 4, 6, and 8 compared to those at 72 h and at week 8 regarding the week. RV WT was significantly higher at weeks 4, 6, and 8 compared to 72 h. PAB animals’ RV systolic function echocardiographic variables (TAPSE, RV FAC, and S-wave (S’) did not present significant differences among the several assessment moments BAP.

Among the diastolic function parameters of the RV, PAB resulted in a higher E/E ratio at 72 h, 6 weeks, and 8 weeks compared to the control subjects (Figure 4E). PAB animals presented a higher E/E ratio at 72 h compared to 4 weeks and in the comparison between 6 and 2 weeks.

**FIGURE 5:**
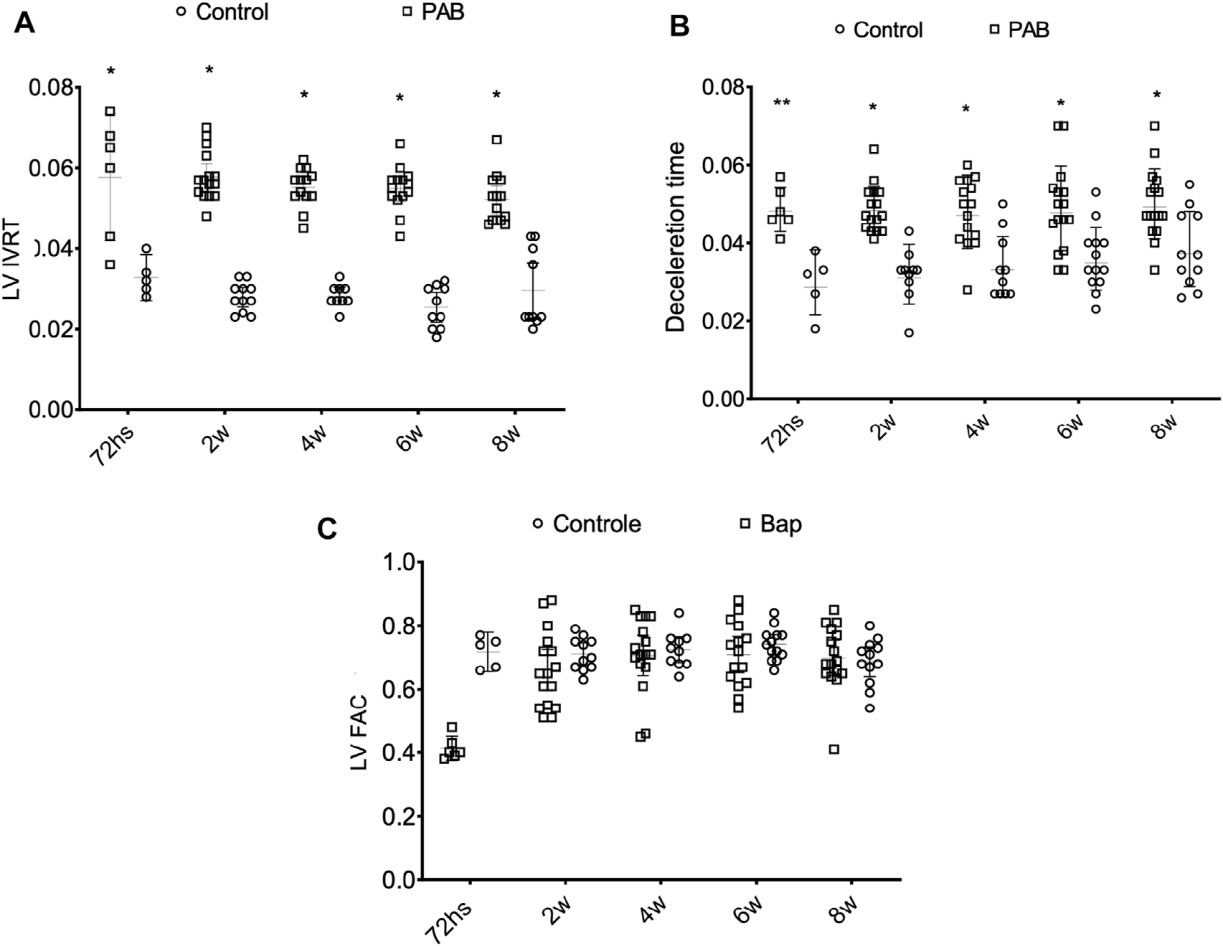
Behavior of the isovolumic relaxation time left ventricle (LV IRVT) **(A)** of the deceleration time **(B)** and of the percent variance of the area of the left ventricles (LV FAC) **(C)** of the PAB animals and control subjects in the different subgroups throughout time. W, weeks; *p < 0.001; **p < 0.01.

In evaluating the parameters in the left heart chambers, we observed that the anteroposterior diameter of the LA, the diastolic and systolic diameters, and the diastolic thickness of the septum and the inferior wall of the LV of the PAB animals were not significantly different from those of the control animals at the different analysis moments. The percent variation of the LV area (LV FAC) was significantly lower in PAB animals than that in the control subjects only in the 72-h subgroup (Figure 5C). Among the PAB animals, this variable was significantly lower at 72 h than that at 2, 4, 6, and 8 weeks. The Doppler study of the LV revealed that the deceleration time of the E-wave and the isovolumic relaxation time (IVRT) was higher in PAB animals in all follow-ups than that in the respective control subjects; however, it did not denote a significant difference among the PAB animal subgroups. Other diastolic function variables of the LV during the echocardiogram did not present significant differences among PAB animals and control subjects (Figure 5).

**FIGURE 6:**
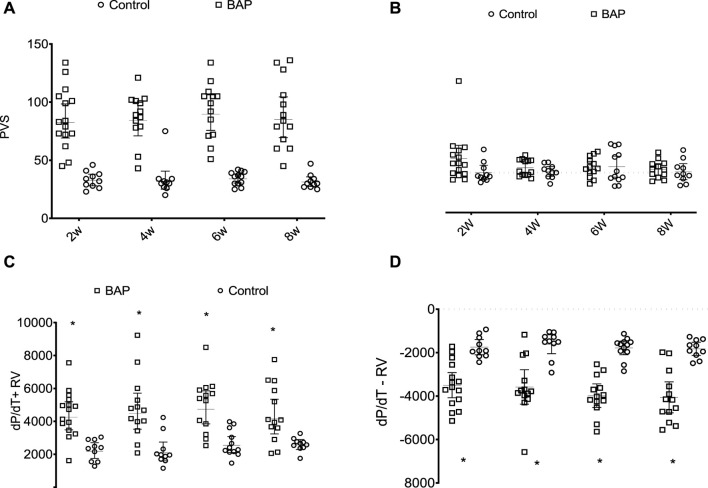
Systolic pressure in right ventricle (PVS, mmHg) **(A)**, final diastolic pressure of the RV (RVEDP, mmHg), **(B)** and derivative of positive (dP/dT + RV) **(C)** and negative pressures (dP/dT-) **(D)** of the RV in PAB animals in comparison to those in control subjects over the different follow-up times. W, weeks; *p < 0.001; **p < 0.01; #*p* < 0.05.

According to the hemodynamic study, the heart rate was not significantly different among the experimental groups (Figure 6). In the RV, systolic pressure values + dP/dT and -dP/dT were higher in PAB animals than those in their respective control subjects. In the LV, diastolic pressure at 6 weeks was significantly higher in the PAB animals than that in the control subjects, and at 6 weeks, +dP/dT was significantly lower in the animals with PAB than that in the control subjects.

Increased gene expression of both ventricles in PAB animals compared to control subjects will be described according to statistical significance (Table 2; Figure 6). The determined function was considered to present increased expression when the number of altered genes is at least 50% of the genes in such a group (Table 3).

**TABLE 3:** Genes with significant increased expression in the RV in relation to the control subjects at each one of the PAB follow-up times in accordance with the exercised cellular function.

Gene function	72 h	2 weeks	4 weeks	6 weeks	8 weeks
Extracellular matrix	col3a1	col3a1 and col1a1	col1a1	col1a1 and Map3k2	Map3k2 and Mnp9
Hypertrophy	Nppa and Nppb	ACE, Nppa, and Nppb	Edn1	IGF 1, Agr1a, Ace2, and Prkcg	None
Angiogenesis		None	None	None	None
Oxidative stress	None	None	CAT and Hspa1a	GPX4, CAT, and Sod1	Sod1
Apoptosis	None	HK1 and AKt1	HK1	Bax, AKt1, Mapk14, Fas, and HK1	Bax, Mapk14, and Mapk1
Calcium kinetics	Atp2a2 and Slac8a1	Atp2a2, Slac8a1, and Casq2	Slac8a1	Atp2a2, Ryr 2, Slac8a1, and Casq2	Pin and Atp2a2
Cellular metabolism	Tp53, Ucp2, and Ndufa3	Tp 53 and Ucp2	Tp 53, Ucp2, Shamlc2a1, and Ucp2	Ndufa3	Ndufa3 and Shamlc2a1
Fetal phenotype	Myh6	Myh6	Myh7	Myh7	Myh7

RV gene expression assessment was performed in 25 PAB animals and 25 control animals (Figure 1). After 72 h of PAB, in comparison to the control subjects, a significant increase in the RV (*p* < 0.01 to 0.001) was observed from the gene activity related to several functions, except for those related to angiogenesis, oxidative stress, and apoptosis (Table 3). After 2 weeks of PAB, a significant increase (*p* < 0.05 to 0.001) was observed regarding the gene activity control subjects related to the same functions observed at 72 h, with the addition of the genes related to apoptosis and angiogenesis (Table 3). At 4 weeks of PAB, there was no significant increase in the expression of the studied gene activity, except for the gene group related to angiogenesis (*p* < 0.05 to 0.001) (Table 3). At 6 weeks of PAB, a significant increase in gene expression (*p* < 0.05 to 0.001) related to the analyzed cellular functions was observed regarding the control subjects (Table 3). At 6 weeks, in PAB animals compared to the control subjects, a significant increase in gene expression related to hypertrophy and angiogenesis was not observed, but a significant increase in the other analyzed gene expressions was observed (*p* < 0.05 to 0.001) (Table 3).

Table 4 shows a summary of the number of genes per function group that presented significantly greater expression in PAB animals than control subjects. The numbers marked in red indicate the condition in which 50% or more of the genes of the group presented a significant increase, which were hypertrophy at 2 weeks, oxidative stress at 4 and 6 weeks, apoptosis at 6 and 8 weeks, calcium kinetics at 2 and 6 weeks, fetal phenotype in all analyzed groups, and inflammation at 72 h, and 2 and 8 weeks (Table 4).

**TABLE 4:** Number of genes with significantly increased expression in each function in the RV in each follow-up time subgroup of PAB animals.

Function	N	72 hs	2 w	4 w	6 w	8 w
Extracellular matrix	7	1	2	1	2	2
Hypertrophy	13	1	7	1	5	0
Angiogenesis	1	0	0	0	0	0
Oxidative stress	4	0	0	2	3	1
Apoptosis	6	0	2	1	5	3
Calcium kinetics	5	2	3	1	4	2
Metabolism	6	2	1	2	2	2
Fetal phenotype	2	1	1	1	1	1
Inflammation	3	2	1	1	1	3

N, total number of studied genes in each function; hs, hours: w, weeks. The groups with over 50% of increased expression in genes are represented in red.

The gene expression analysis in the LF was conducted in 20 PAB animals and 20 control animals at all follow-up times, except in the 72-h group (Figure 1). Following 2 weeks of PAB, there was significantly increased activity of the genes related to the extracellular matrix, hypertrophy, oxidative stress, and apoptosis compared to the control subjects (Table 4). After 4 weeks of PAB, there was a significant increase in gene expression in all functions except for inflammation-related gene expression.

Table 5 shows at 6 weeks of PAB, in the LV, there was a significant increase only in the gene related to inflammation, and at 8 weeks, there was a significant increase in gene expression in all functions except angiogenesis (Table 4).

**TABLE 5:** Significantly increased gene expression in the LV compared to the control subjects in each PAB follow-up time and according to the exercised function.

Gene function	72 h	2 weeks	4 weeks	6 weeks	8 weeks
Extracellular matrix	NR	col3a1, Tgf, and col1a1	col3a1, Tgf, col1a1, and Mnp9	Gapdh VE	Gapdh VE, col3a1, and col1a1
Hypertrophy	NR	ACE: Nppa, Nppb, and Agr1a	IGF1, Nppa, Nppb, Ace2, Cabin1, and Edn1	None	Prkca, Ace2, Prkcb, and Edn1
Angiogenesis	NR	None	Vegfa	None	none
Oxidative stress	NR	HShampa1a	HShampa1a and Shamod1	None	GPX4 and Shamod1
Apoptosis	NR	Bax	Bax, FaSham, and Mapk1	None	FaSham, HK 1, and AKt1
Calcium kinetics	NR	None	CaShamq2	None	Pin, Slac8a1, Atp2a2, Casq2, and Ryr2
Cellular metabolism	NR	None	Tp 53 and Ucp2	None	Ucp2, Ndufa3, and Shamlc2a1
Fetal phenotype	NR	None	Myh7	None	Myh7
Inflammation	NR	None	None	IL-6	IL-6

NR, not realized.

Table 6 shows a summary of the number of genes per function group that presented significantly greater expression in PAB animals than in the control subjects. The numbers marked in red indicate the condition in which 50% or more of the genes of the group presented a significant increase in extracellular matrix at 2 and 4 weeks, angiogenesis at 4 weeks, oxidative stress and apoptosis at 4 and 8 weeks, calcium and metabolism kinetics at 8 weeks, and fetal phenotype at 4 and 8 weeks (Table 5).

**TABLE 6:** Number of significant increases in expression in the LV in each time subgroup in PAB animals.

Function	N	2 s	4 s	6 s	8 s
Extracellular matrix	7	4	6	0	3
Hypertrophy	13	4	6	0	4
Angiogenesis	1	0	1	0	0
Oxidative stress	4	1	2	0	2
Apoptosis	6	1	3	0	3
Calcium kinetics	5	0	1	0	5
Metabolism	6	0	2	0	3
Fetal phenotype	2	0	1	0	1
Inflammation	3	0	0	1	1

s, weeks. The groups with over 50% of increased expression in genes are represented in red.

## 4 Discussion

The animal model used efficiently produced significant pulmonary artery stenosis (Rockman et al., 1994), with a mortality rate of 19.9%. Models with isolated pressure overload would have lower mortality rates and the development of heart failure concerning the models that mimic pulmonary hypertension (Bogaard HarmJ. et al., 2009; Bogaard et al., 2009b; Bogaard et al., 2009c; Bogaard et al., 2009c; Voelkel et al., 2011). Higher levels of fibrosis in the RV, with the decrease of capillary density and reduction in the remodeling factors of oxidative stress, and the reduction of NRF2 and its targeted gene HO-1, would be determined to have a higher incidence of heart failure (Bogaard et al., 2009b).

In this study, pulmonary artery stenosis resulted in expressively high-pressure gradients accompanied by a systolic pressure increase in the RV, contributing to the study of hemodynamic and structural and morphological repercussions. However, the isolated pressure overload did not determine right or left heart failure, as assessed by total weight and dry weight of the liver and lungs, in contrast to what was observed in previous studies (Bogaard et al., 2009b; Bogaard et al., 2009c). The evolution into heart failure in patients with pulmonary hypertension and RV overload in humans varies. Some patients rapidly develop a heart dysfunction, and others present the same condition but endure without heart failure (Herron and McDonald, 2002; Voelkel et al., 2011). The most comprehensive hypothesis reveals that the interaction of the neuro–hormonal mechanisms—oxidative stress, inflammation, ischemia, and those of programmed cell death (apoptosis)—present in the condition of pressure overload are intensified by the persistence of stimuli and determine the installation of right ventricular insufficiency.

Structural changes that determine the myocardial maladaptation of the RV were observed in the studied model, and a follow-up time longer than 8 weeks could illustrate the accentuation of this frame with the right IC installation potential. The signals of right cavity remodeling were observed in the morphological study and the echocardiogram. There was a significant increase in the right atrium weight and the RV weight, and AD area 2, 4, 6, and 8 weeks after PAB in relation to the control subjects and to every subgroup follow-up time analyzed. The increase in weight of the RV of the PAB animals is likely due to the increased myocardial thickness (Gottdiener et al., 1985) since the echocardiogram showed that the diastolic diameter was only significantly higher than that of the control group of 8 weeks of PAB. In contrast, the myocardial thickness was significantly higher at 4 weeks.

The histological analysis denoted the adjustment process of the RV, with a significant increase in the volume of the myocyte nucleus at all follow-up times, with higher values at 72 h and 2 weeks, simultaneously with the gene expression. Elevation was associated with hypertrophy for 2 weeks. Such findings of higher levels of hypertrophy reached during the earliest moments contradict the concept that the longer the PAB follow-up time, the greater the hypertrophy would be (Gottdiener et al., 1985). However, the increased nuclear volume denotes an increase in the cellular activity soon after PAB to produce hypertrophy and adjust to the pressure overload condition.

There was increased myocardial fibrosis in the animals at 4, 6, and 8 weeks of PAB follow-up time compared to control subjects, followed by a greater number of genes with significant expression for apoptosis at 6 and 8 weeks. In these same subgroups, significant changes in the markers of systolic function of the RV were noticed on the echocardiogram results. VPAVD and the S-probe were lower than the control subjects at the follow-up times of 2, 4, 6, and 8 weeks, coincident with fibrosis at 4, 6, and 8 weeks, indicating incipient systolic dysfunction despite the non-variation of the systolic function parameters of the RV to the hemodynamic study, which denoted the preserved capacity of generating strength even with increased dp/Dt compared to control subjects.

The RV’s diastolic function should be interpreted in light of the comparison with the control group and in parallel with the available data for the left ventricle. There is scarce data analysis on the diastolic function of the RV to perform an echocardiogram in the literature (Laks et al., 1972). As per the guideline, at least three parameters should be altered to diagnose diastolic pressure dysfunction or increase: increased volume of the left atrium, increased pulmonary systolic pressure, reduction of septal or lateral E waves, and an increased E/E’ ratio (Nagueh et al., 2016). In the present study, we must consider that there is already an established myocardial alteration in PAB. From the diastolic pressure increase indicating parameters, we can safely consider that in animals with PAB, the systolic pressure of the RV was increased, for there was an increase in the right atrium and E/E ratio at 72 h, and 6 and 8 weeks. Thus, among the four analyzed parameters, three were altered; therefore, we can consider that according to the echocardiogram, there was increased diastolic pressure, coincident with the data in the literature (Horton et al., 2009; Rudski LG. et al., 2010). This elevation of the E/E ratio and the RV concurred with a greater number of increased expression genes and was related to calcium kinetics and oxidative stress. It is worth considering, however, that the parameters obtained from the invasive study denoted preserved diastolic pressures in PAB animals, and the negative dP/dT was even significantly higher in PAB animals, which suggests greater relaxation of the RV. Nevertheless, such variables may not reflect the myocardial performance as it has already been observed in hypertrophy models of the LV with pressure overload by beta-adrenergic stimulation. In this study, contraction and relaxation of the papillary muscle were decreased, reflecting the altered myocardium but with preserved systolic performance (Serra et al., 2010).

In the evaluation of the gene expression related to the fetal phenotype, there is a transition from the adaptive pattern to the maladaptation phase, which has already been reported in several injury models, occurring from a change in the pattern of standard light myosin chains α (MHC–α) to standard heavy myosin chains β (MHC–β) in cardiomyocytes (Bogaard et al., 2009c; Egemnazarov et al., 2015). In humans, the right ventricle has 25%–34% MHC-α, and the remaining is MHC-β. MHC-β has lower ATP activity than MHC-α; therefore, a decrease in the alpha chain would contribute to a decrease in systolic performance (Herron and McDonald, 2002). In the present study, the predominance of MHC-α occurred at early follow-up times of 72 h and 2 weeks, and the beta pattern was present in the groups of 4, 6, and 8 weeks, which coincided with the follow-up times of the emergence of fibrosis in the histology and the decrease in the RV function on the echocardiogram. The correlation of the emergence of fibrosis with decreased systolic function is known in the literature (Leeuwenburgh et al., 2003; Leeuwenburgh et al., 2003; Faber et al., 2006; Bogaard et al., 2009b), and it was observed in the RV of the present study with the concomitant observation of the change in the gene expression pattern to beta. The inflammation genes had increased expression at all PAB follow-up times, with more genes at the follow-up times of 72 h and 8 weeks, reflecting the most acute state of injury and the one with the longest follow-up time exposure.

Alterations in the RV were followed by changes in the LV, with an increased volume of the nucleus of the cardiomyocytes, which had already been observed in other experimental studies (Laks et al., 1972; Gomez et al., 1993). In human beings, the alternate situation has already been observed, namely, hypertrophy of the myocardial fibers of the RV in diseases such as aortic stenosis and systemic arterial hypertension, which may develop with RV hypertrophy (Krayenbuehl et al., 1989; Amanuma et al., 1994). In these reports and in this study, the severity of the condition is correlated with the intensity of hypertrophy and fibrosis in both ventricles, and such data confirm the thesis of the interdependence of ventricular chambers, which, despite having distinctive embryologic origins, are submitted to the same hemodynamic, humoral, and neural stimuli and, therefore, present only answers that are concomitant. LV fibrosis was described in Tetralogy of Fallot patients (ChenDusenbery et al., 2016; Zile and Gregg, 2016). There is a report of a meaner prognostic in situations of left pressure overload at the emergence of fibrosis (Hein et al., 2003). In the model presented here, there was progressively increasing fibrosis in the LV in PAB animals, although with significant differences compared to the control subjects at weeks 2 and 8.

The echocardiogram’s anatomic assessment of the left cardiac cavities in PAB animals revealed no expressive changes. Regarding the systolic function, PAB animals had a significantly lower value of VPAVE at 72 h compared to the control subjects. Possibly, there was a sharp and intense change in the RV geometry immediately after PAB, which may have induced a transitory maladaptation of the LV. Such a variation did not occur in the other subgroups in the echocardiographic analysis. The hemodynamic data on the LV systolic function illustrate that + dP/dT in PAB animals was significantly lower than that in control subjects at only 6 weeks. At other analysis follow-up times, +dP/dT was lower in the PAB group but without a significant difference. Perhaps other non-invasive analysis methods, such as the myocardial deformation detected by an echocardiogram, could detect the nuance of change observed in the invasive hemodynamic study. Among the diastolic function parameters of the LV, just TRIV and TDE were altered in PAB animals. However, the size of the left atrium assessed by the anteroposterior diameter was not significantly altered in PAB animals compared to the control subjects, and the E-wave and, subsequently, the E/E ratio were not analyzed. Thus, the diastolic function analysis of the LV through the echocardiogram was limited, and it does not enable accepting the existence of diastolic dysfunction. The TRIV and TDE changes observed were also described in other studies (Laks et al., 1972; Noordegraaf AntonVonk et al., 1997). As to the invasive study, it was observed that PDVE was higher in the animals with PAB than in the control subjects at 6 weeks.

Other researchers have also documented alteration in the gene expression in the LV as a consequence of PAB (Ikeda et al., 1999; Braun et al., 2003). Gene expression related to the extracellular matrix, hypertrophy, angiogenesis, oxidative stress, apoptosis, and increased expression of MHC-β occurred more intensively at 4 and 8 weeks, simultaneously with the increased weight of the LV. The expression of the genes related to cellular metabolism and calcium kinetics in the LV was altered only at 8 weeks of PAB.

These results suggest the effect of RV hypertrophy on the LV, as detected by the histologic and genetic changes, with no significant functional impairment of the LV until at least 8 weeks of PAB.

Alterations of the LV in association with the impairment of the RV have already been denoted in studies on rabbits subjected to PAB, in which diastolic dysfunction of the LV was reported with preserved ejection fraction and an increased myocardial performance index (Kitahori et al., 2009). A study on human beings bearing CPOD examined with magnetic resonance imaging denoted a decrease in the LV function compared to their control subjects (Noordegraaf AntonVonk et al., 1997).

Some limitations to this study should be pointed out. A longer analysis period, 12 weeks of PAB, could culminate in heart failure. In the literature, studies published up to this moment had a follow-up of various time lengths ranging from 3 to 8 weeks (Kitahori et al., 2009; Egemnazarov et al., 2015), and heart failure was not detected either. However, studies of LV overload in rats at 15 weeks of aortic bandage resulted in clear IC signals (Dolgilevich et al., 2001). Another limitation was that gene expression analysis of the LV and hemodynamic studies was not performed 72 h after PAB and their control subjects as a consequence of the technique restriction for the conduction of the experiments.

## 5 Conclusion

Rats subjected to PAB presented RV remodeling compatible with hypertrophy. Such alterations were measured by increased gene expression and functional changes coinciding with the emergence of fibrosis. The structural variables of the RV, such as weight, myocardial thickness, myocyte nuclear volume, and fibrosis degree, were modified according to the exposure time to PAB, and they had a relationship with the variations in gene expression, highlighting the change from an alpha to a beta pattern from initial times to late times. The study suggests that the LV developed histologic changes, followed by modifications in gene expression simultaneously with the alterations found in the RV (Johns and Olson, 1954; Nogueira et al., 1991; Bauer et al., 1998; TOYODA et al., 1998; Bjornerheim et al., 2001; Farahmand et al., 2004; Dai and Isoyama, 1993; Buermans et al., 2005; Molina et al., 2009; He et al., 2012; Brown et al., 2013; Friedberg et al., 2013; Lu et al., 2013).

## Data Availability

The original contributions presented in the study are included in the article/Supplementary Materials; further inquiries can be directed to the corresponding authors.
