# Supplementary material for: Hypertrophy of the right ventricle by pulmonary artery banding in rats: a study of structural, functional, and transcriptomics alterations in the right and left ventricles
Source: Front Physiol. 2023 Jul 27;14:1129333. doi: 10.3389/fphys.2023.1129333 (PMC10414540; doi:10.3389/fphys.2023.1129333)

**Supplementary material**

Data sheet 2

Annex 2

Molecular biology data of the right ventricle in heat maps by time groups

1. 72hs right ventricle


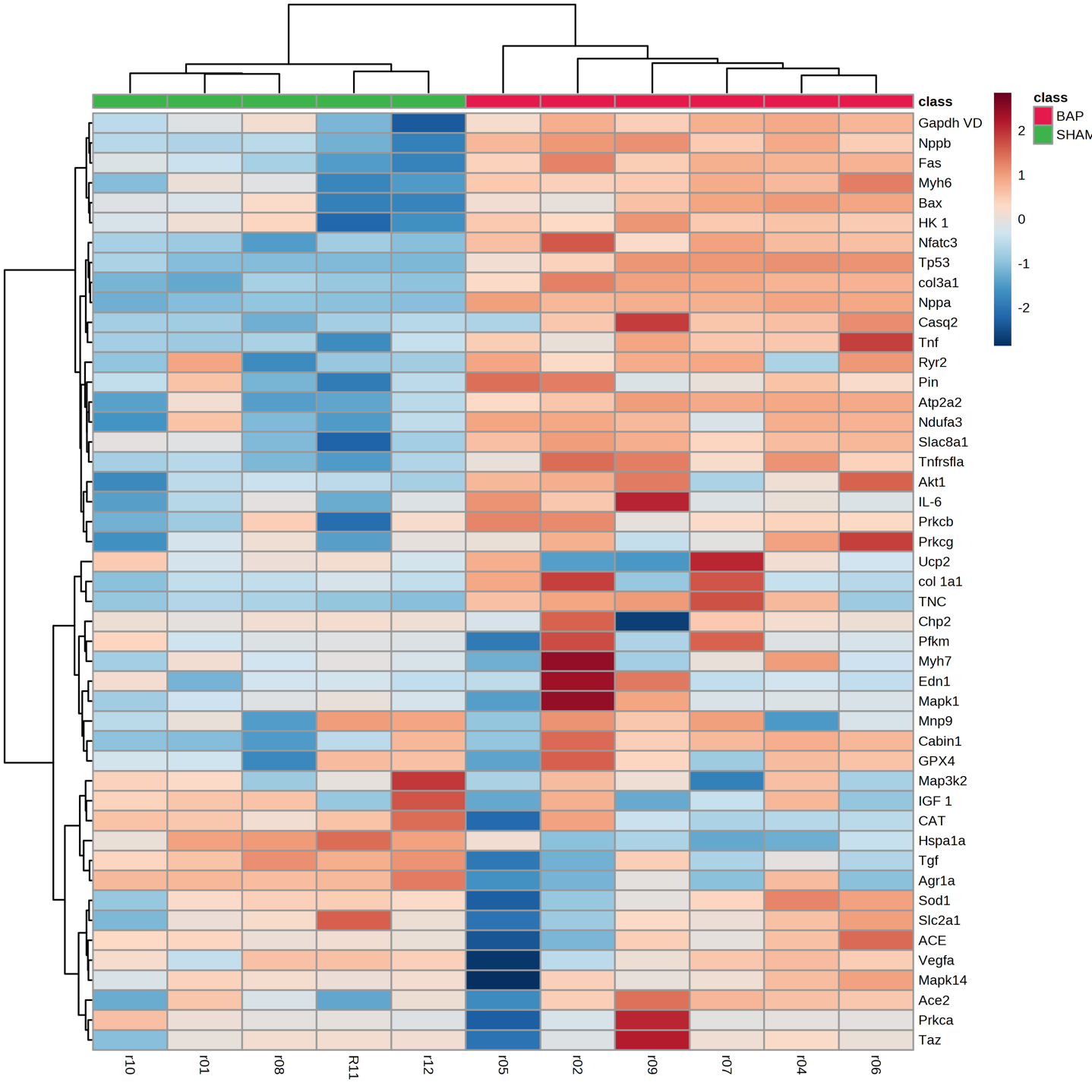


1. 2 weeks- right ventricle


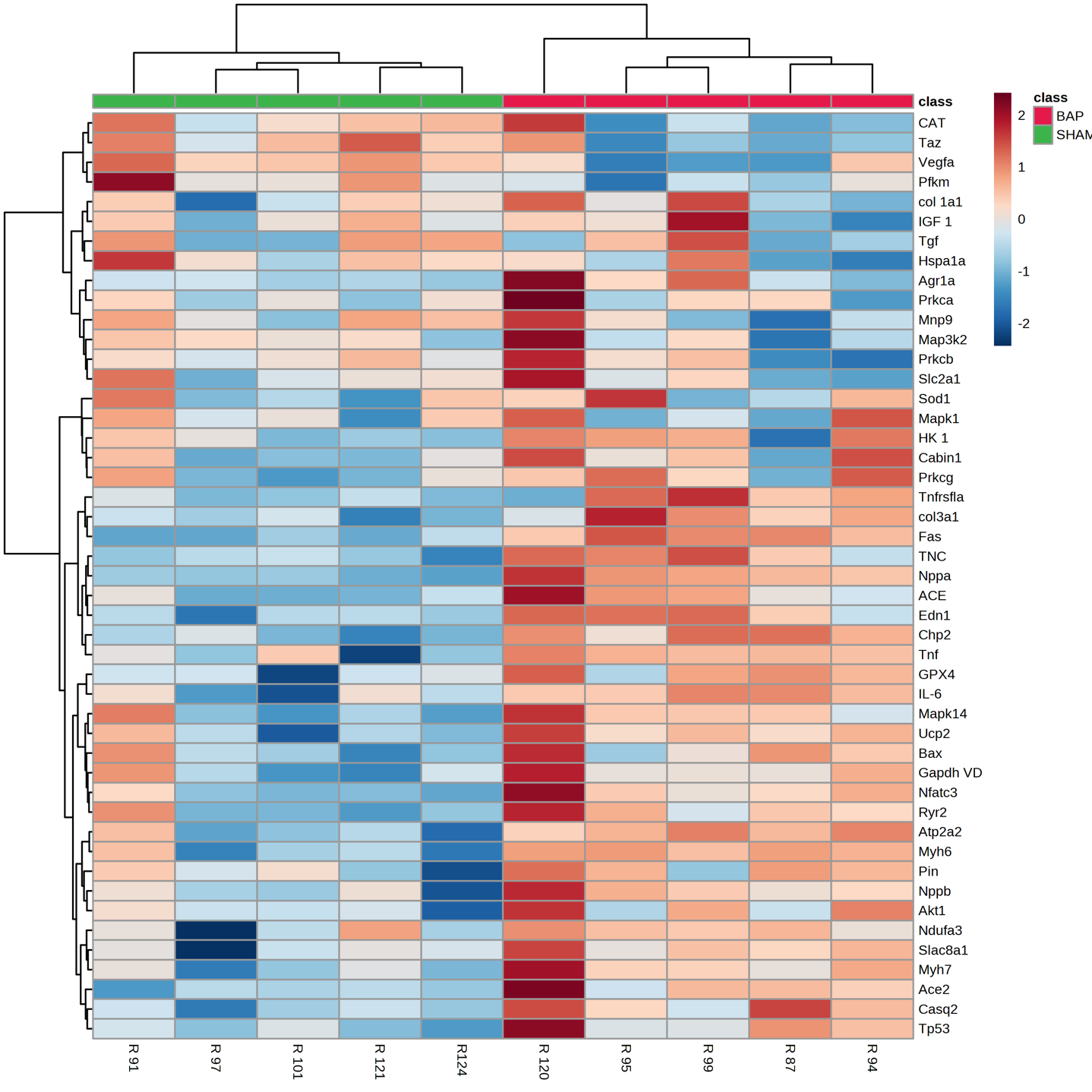


1. 4 weeks- right ventricle


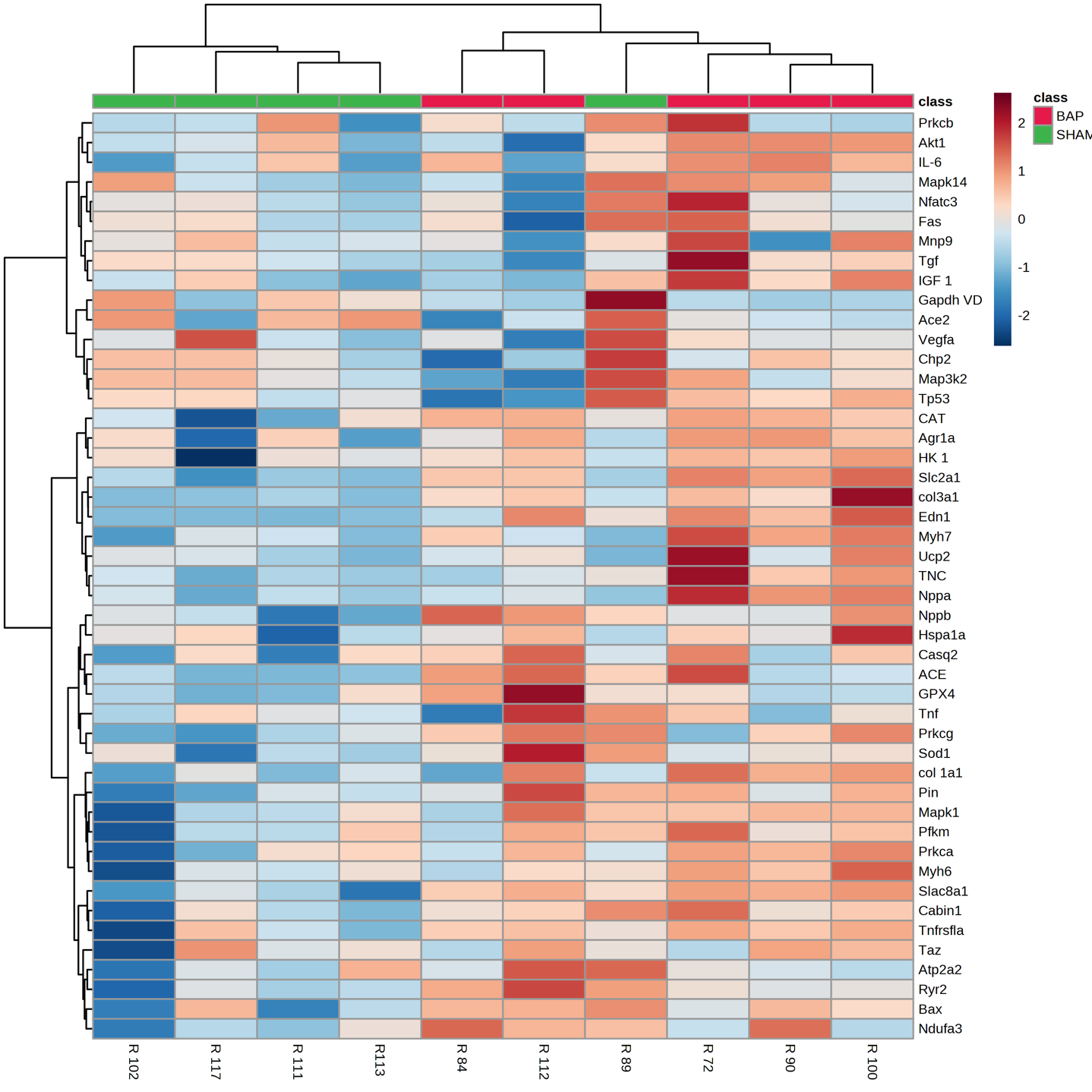


1. 6 weeks- right ventricle


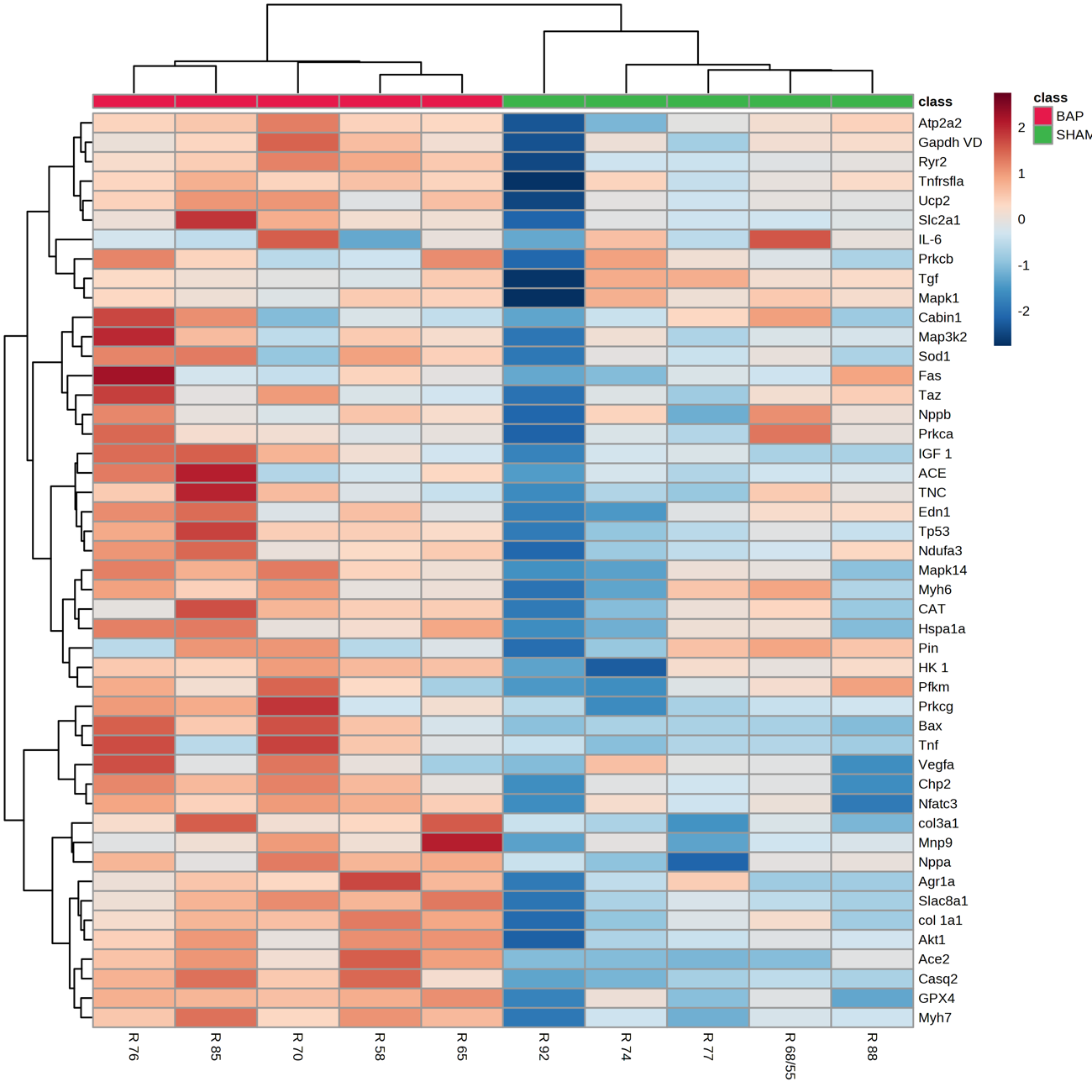


1. 8 weeks- right ventricle


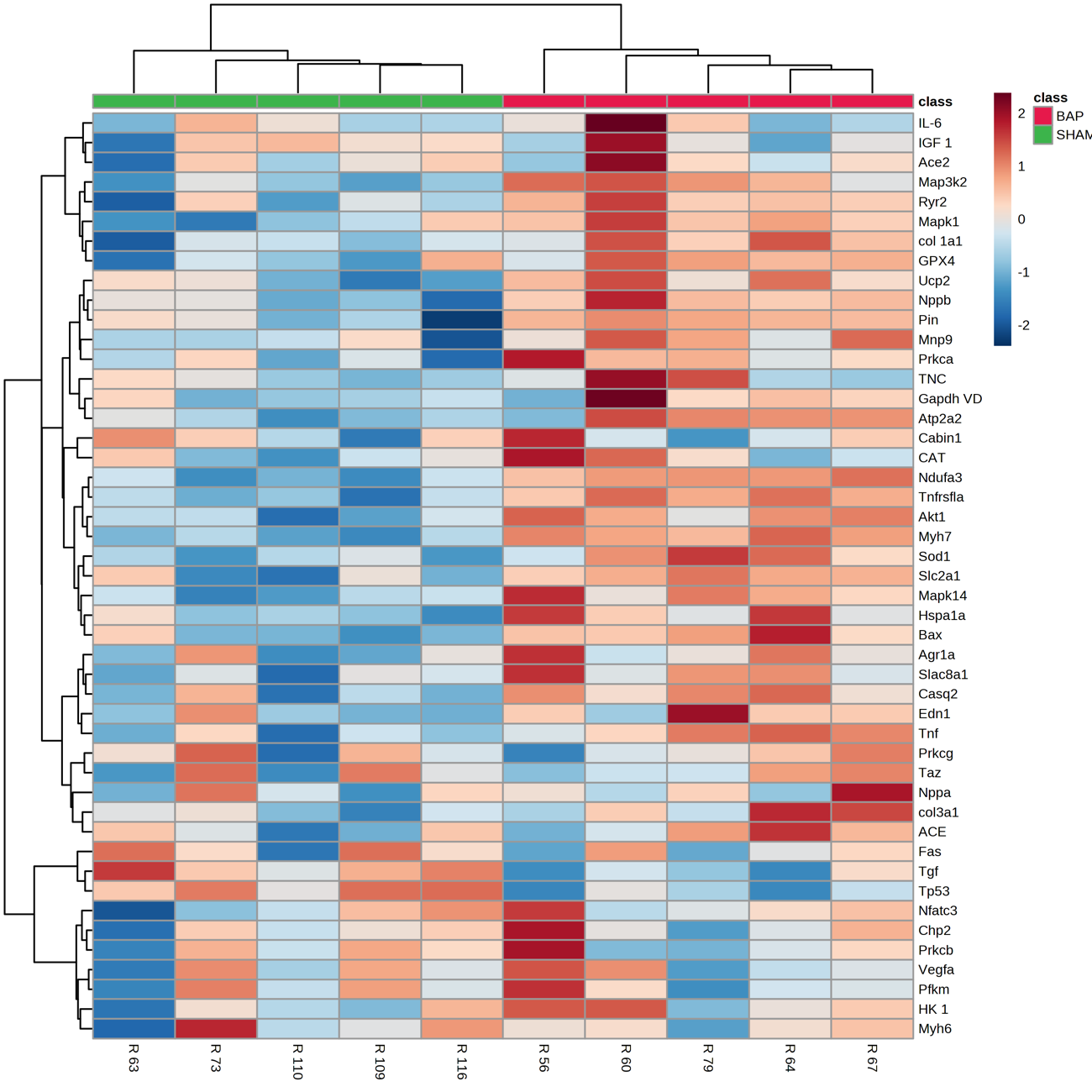

Supplement: Supplementary file 1 [file DataSheet2.docx]
