# Supplementary material for: Hypertrophy of the right ventricle by pulmonary artery banding in rats: a study of structural, functional, and transcriptomics alterations in the right and left ventricles
Source: Front Physiol. 2023 Jul 27;14:1129333. doi: 10.3389/fphys.2023.1129333 (PMC10414540; doi:10.3389/fphys.2023.1129333)
Supplement: Supplementary file 2 [file DataSheet1.docx]

Supplementary material

Data sheet 1

Annex 1: Echocardiographic analyses

For morphological and functional analysis of the heart several measurements were performed, and various parameters calculated according to previous experience of our laboratory and international guidelines with adaptations for the animals of the present study (12,19,21,22,23,24,25,38).

**Atrium**

The size of the left atrium was evaluated by the anteroposterior systolic diameter measured in the longitudinal parasternal plane in the two-dimensional image or preferably in the M mode stroke, expressed in mm. The size of the right atrium was evaluated by the systolic area measured in the apical plane 4-chambers with the planimetry and expressed in cm2 (Figure 1).

 

Figure 1. apical image showing the 4 cardiac cavities; in systole (A) used to measure the right atrium; B: in diastole to measure the thickness and diastolic diameter of the RV

**Right ventricle**

In the apical four-chamber image, the basal transverse diastolic diameter (in mm) was measured as the distance from the septum to the lateral wall and also the diastolic thickness of the free wall (figure 2 B). Three parameters were obtained for the analysis of systolic function: shortening fraction of the area, systolic excursion of the tricuspid ring and displacement speed of the tricuspid ring. Shortening fraction (SHF) as Ad-As/Ad x 100 (Figure 2), expressed as a percentage (%) based on measurements of the diastolic (Ad) and systolic (As) areas in the longitudinal 4-chamber plane. The systolic excursion of the lateral tricuspid valve annulus (ESAT) toward the apex was obtained on the apical 4-chamber image with the M-mode ultrasound beam aligned parallel to the RV free wall and perpendicular to the lateral plane of the tricuspid valve annulus; The systolic excursion of the tricuspid annulus was measured as the distance from base to peak excursion.


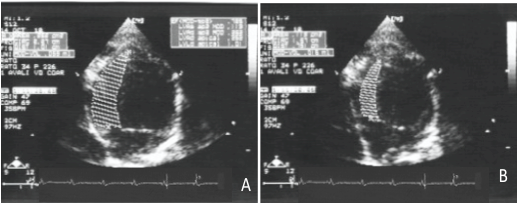


Figure 2. Image in the apical 4-chamber plane demonstrating the measurement of the diastolic area (A) and systolic (B) used to calculate the percentage change of RV area.

            For the analysis of RV diastolic function, the E and A waves (in cm/s) were measured in the tricuspid diastolic flow velocity curve (Figure 3A). With tissue Doppler, the initial diastolic velocity of the tricuspid annulus (S-wave, in cm/s) was measured on the apical plane image and the volume sample positioned on the lateral tricuspid annulus (Figure 3 B). Next, with tissue Doppler we measured the e' wave (in cm/s); with these measures, the E/A and E/e' ratios were calculated

.

Figure 3: In A, the diastolic flow velocity tracing with pulsatile Doppler showing waves E and A. In B, the velocity tracing of the displacement of the lateral annulus of the tricuspid valve; note the initial diastolic wave E' and the systolic wave S' used as an index of systolic function.

**Left Ventricle**

  In the transverse or longitudinal parasternal plane, the diastolic and systolic diameters and the diastolic thicknesses of the interventricular septum and LV posterior wall were measured, preferably in the stroke in M mode, or in the two-dimensional image (Figure 4). Systolic function was determined by calculating the shortening fraction of the transverse area (FEA = Ad-As/Ad); for this, the sectional areas were obtained in diastole (Ad) and systole (As) in the transverse plane in the middle region of the cavity (figure 6).


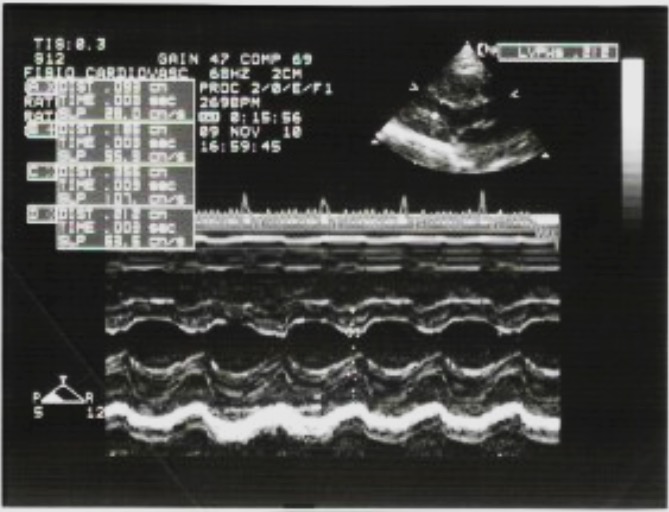


Figure 4. Tracing in M mode of the RV and LV with the ventricular septum between them and the posterior wall. The dotted lines exemplify how the measures were.


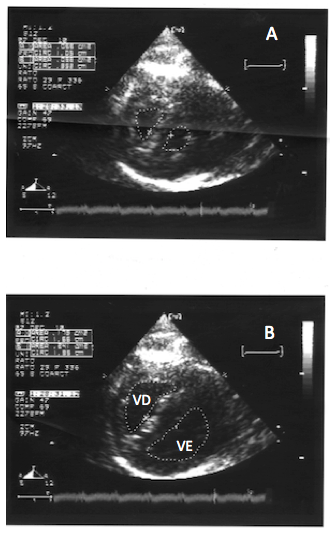


Figure 5. Echocardiographic image obtained in the transverse parasternal plane in the middle region of the right and left ventricles of an animal submitted to pulmonary artery bandage. The dotted lines demonstrate the systolic area of VE in A and diastolic in B . Also note the dilation and increased thickness of the RV free wall with rectified positioning of the ventricular septum.

The diastolic function was analyzed, initially, in the mitral diastolic flow velocity curve, obtained with pulsed Doppler in the apical four-chamber plane with the volume sample positioned on the ventricular side of the mitral valve. On this curve the maximum velocity of the E wave (initial filling) and the atrial contraction (wave A) were measured and the E/A ratio was calculated (figure 6A), and the deceleration time of the E wave. With the pulsed Doppler volume sample positioned between the LV inflow and outflow tracts, the simultaneous flow velocity curve in the two regions was obtained for TRIV measurement (Figure 6B).

Figure 6 A: mitral diastolic flow velocity curve obtained with pulsed Doppler in the in the LV inlet (apical 4-chamber section) for measurement of the E and A waves; B: flow curve obtained between the LV inlet and outlet pathways (LVOT) for measurement of the isovolumetric relaxation time.

**Pressure gradient across the pulmonary artery banding**

It was analyzed with the measurement of systolic flow velocity through PAB with continuous Doppler (Figure 7); the continuous Doppler ultrasound beam was guided with the two-dimensional image and by color flow mapping.

Figure 7: In A, parasternal cross-section at the level of the RV outflow tract and the pulmonary

pulmonary artery trunk; note the color mapping image of the flow through the banding

In B flow velocity curve through lung banding with continuous Doppler used to calculate the systolic gradient.
